# Supplementary figures and images for: Allele-specific enhancer interaction at the Peg3 imprinted domain
Source: PLoS One. 2019 Oct 22;14(10):e0224287. doi: 10.1371/journal.pone.0224287 (PMC6804975; doi:10.1371/journal.pone.0224287)

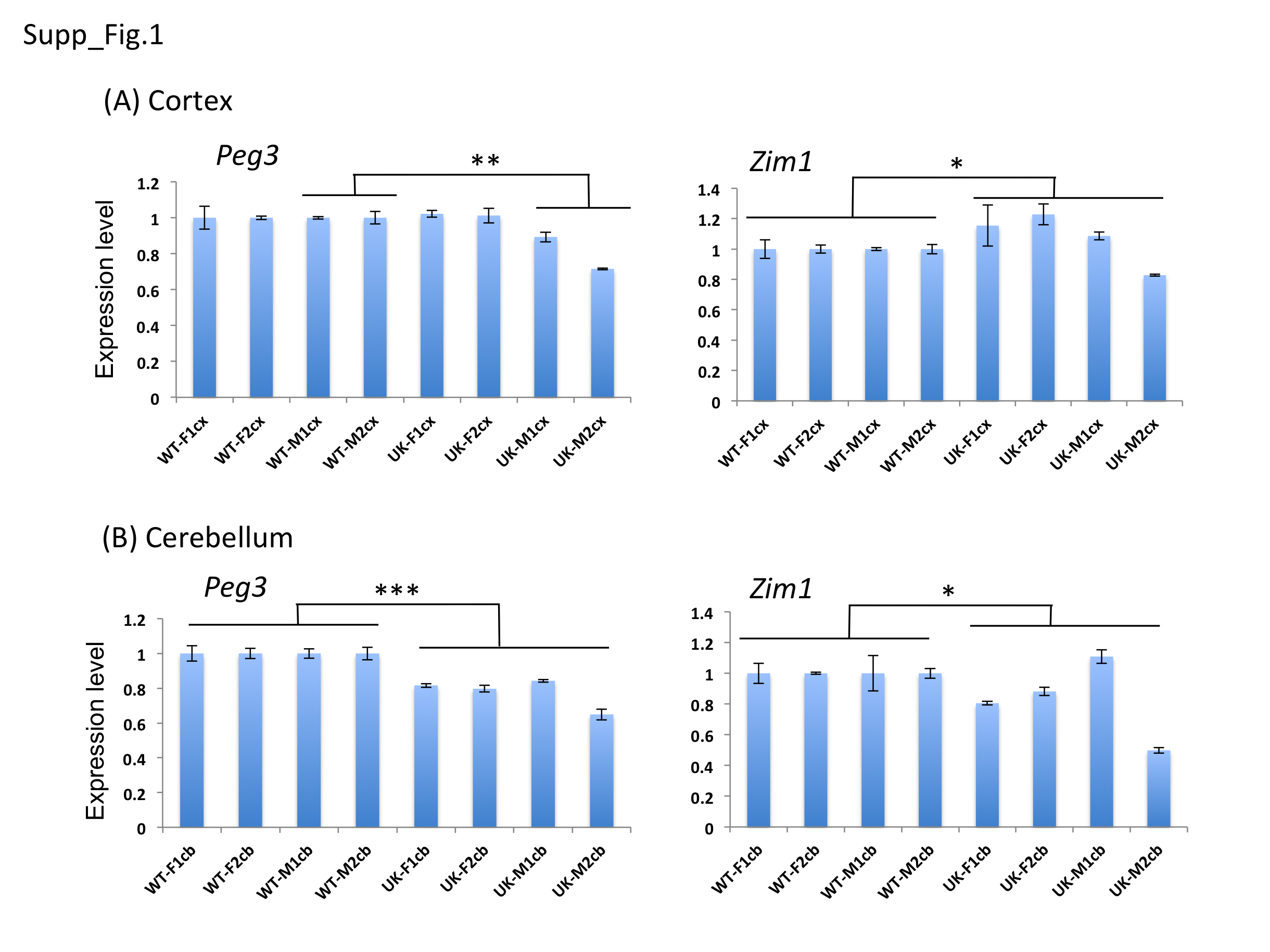

Supplement: S1 File — (TIF) [file pone.0224287.s001.tif]

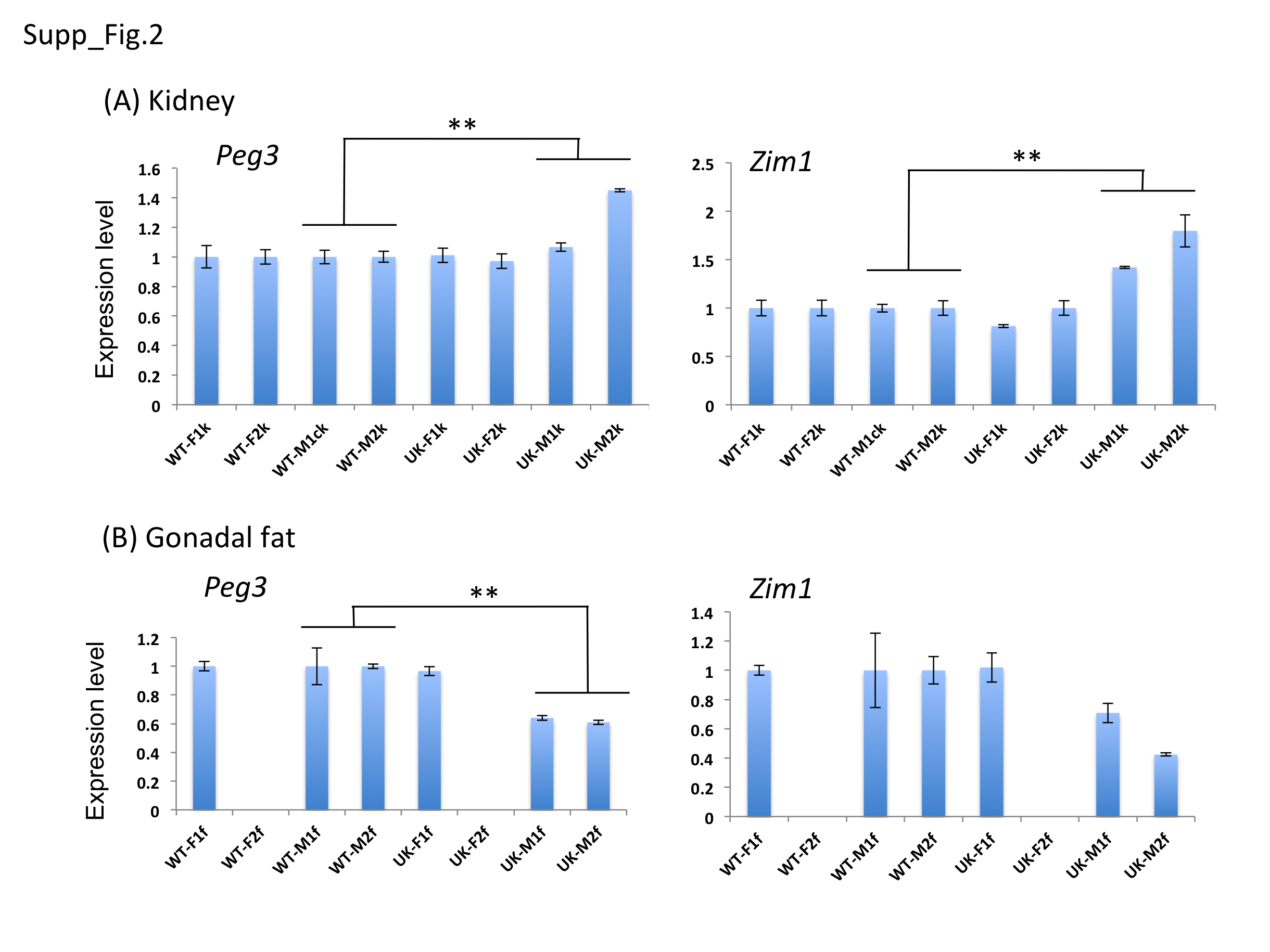

Supplement: S2 File — (TIF) [file pone.0224287.s002.tif]

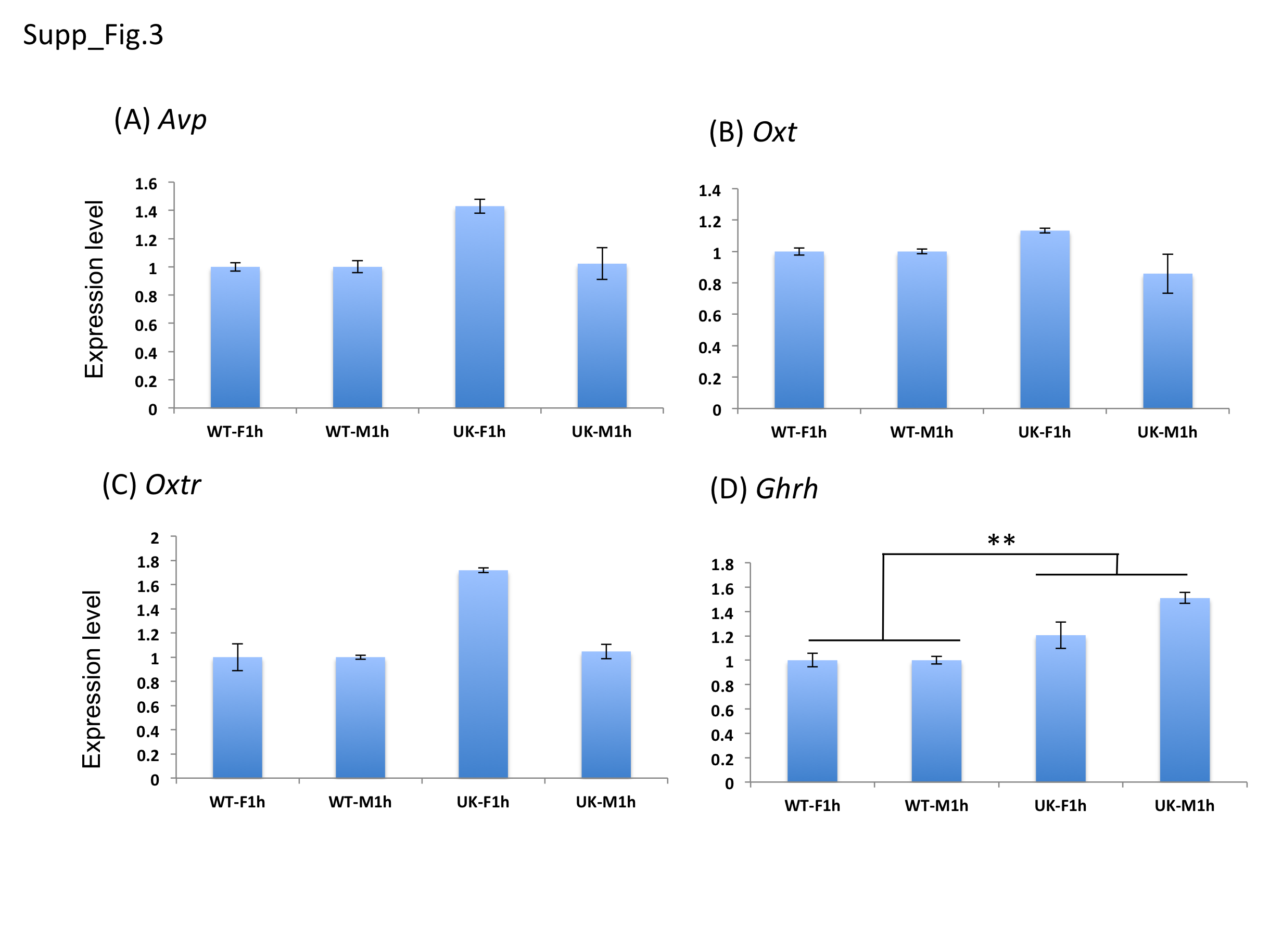

Supplement: S3 File — (TIF) [file pone.0224287.s003.tif]

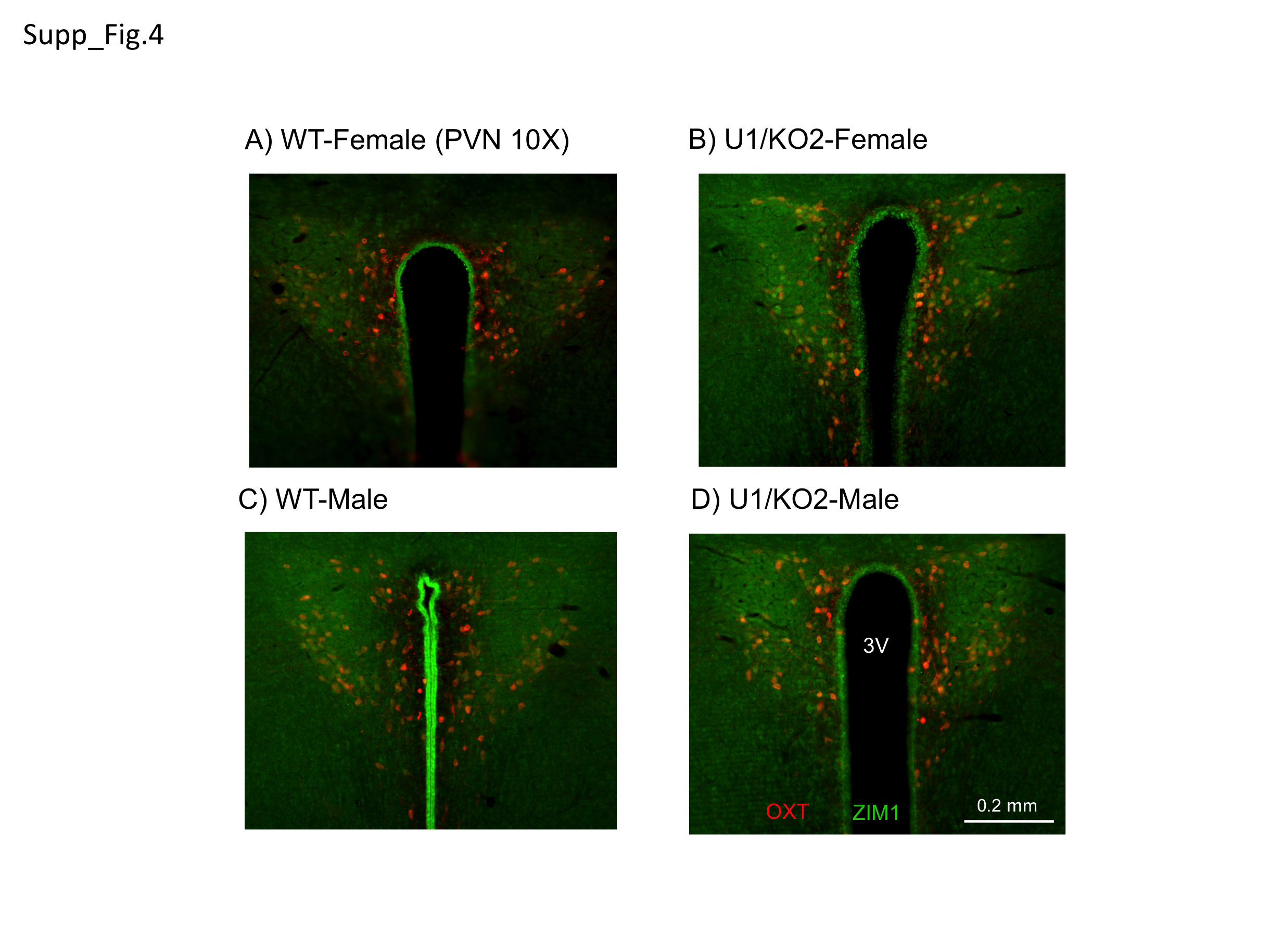

Supplement: S4 File — (TIF) [file pone.0224287.s004.tif]

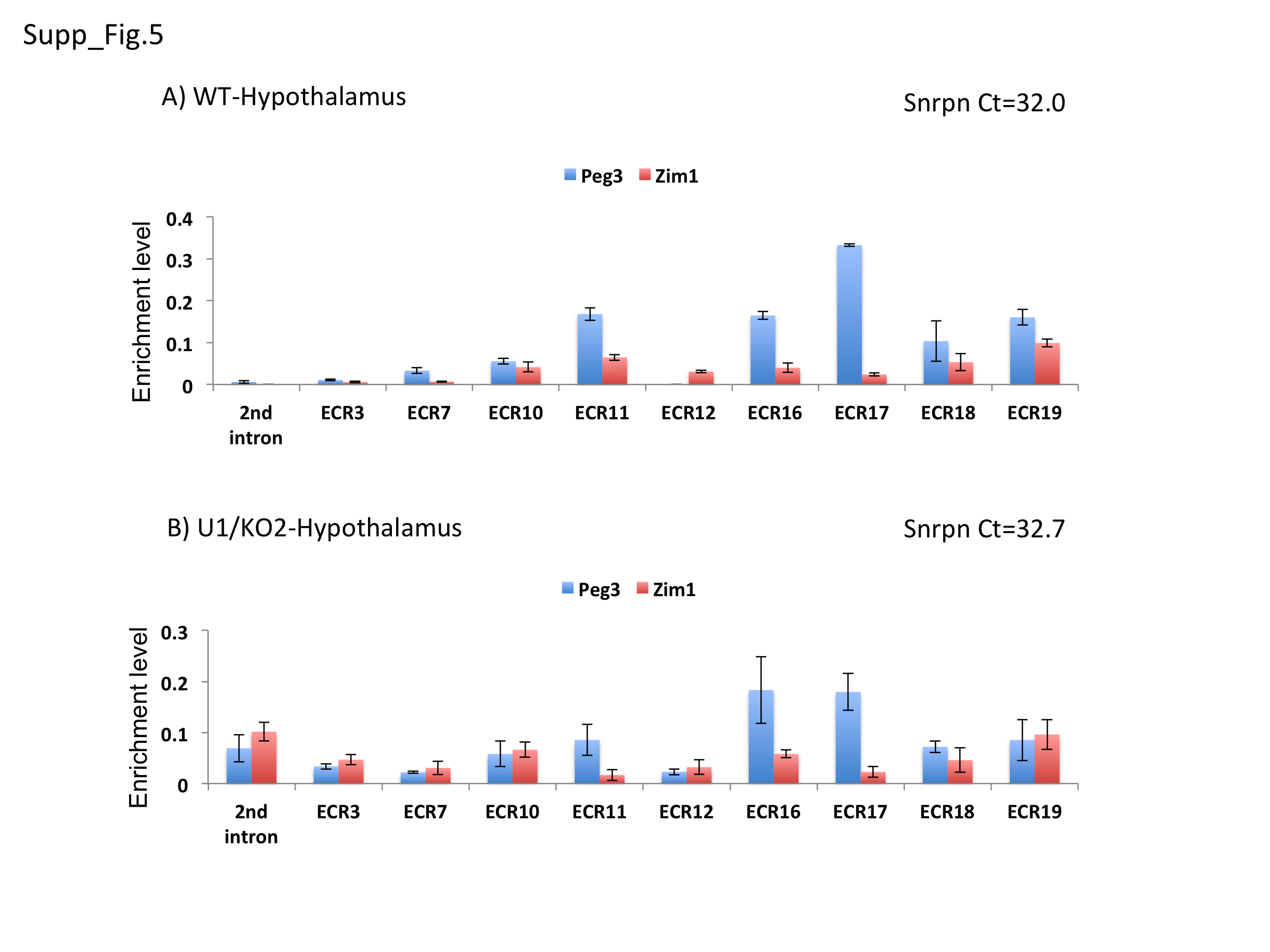

Supplement: S5 File — (TIF) [file pone.0224287.s005.tif]

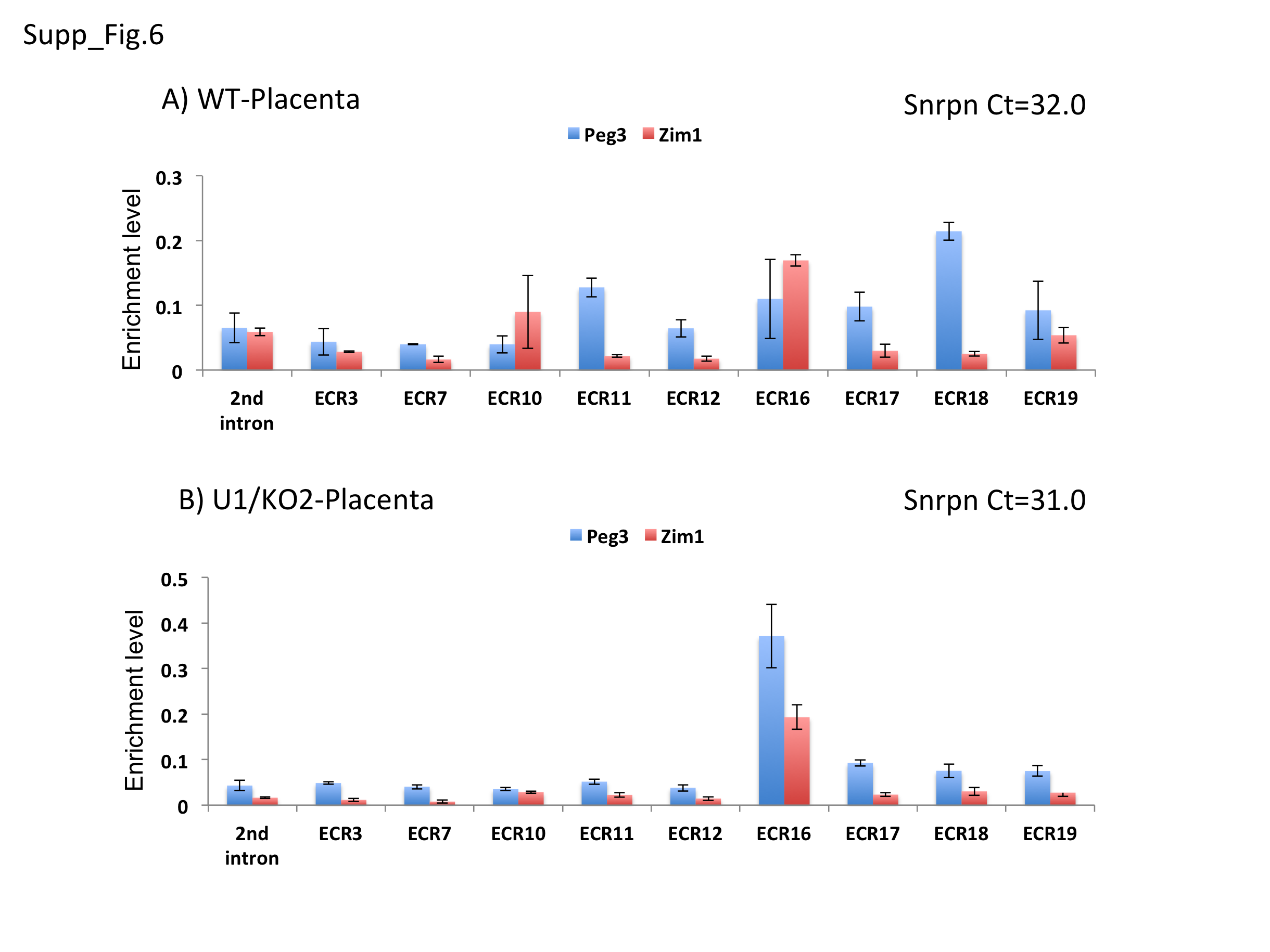

Supplement: S6 File — (TIF) [file pone.0224287.s006.tif]

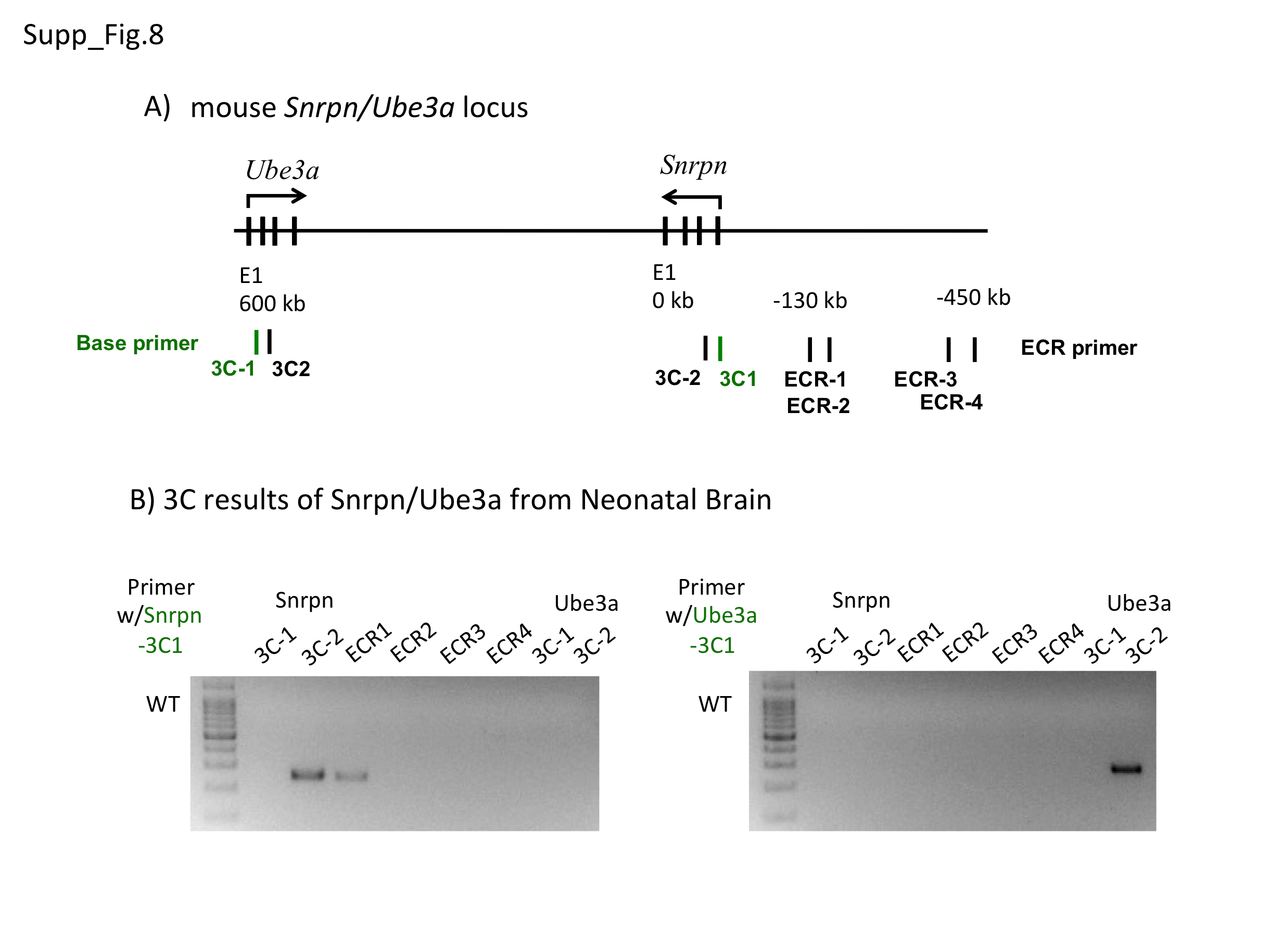

Supplement: S8 File — (TIF) [file pone.0224287.s008.tif]

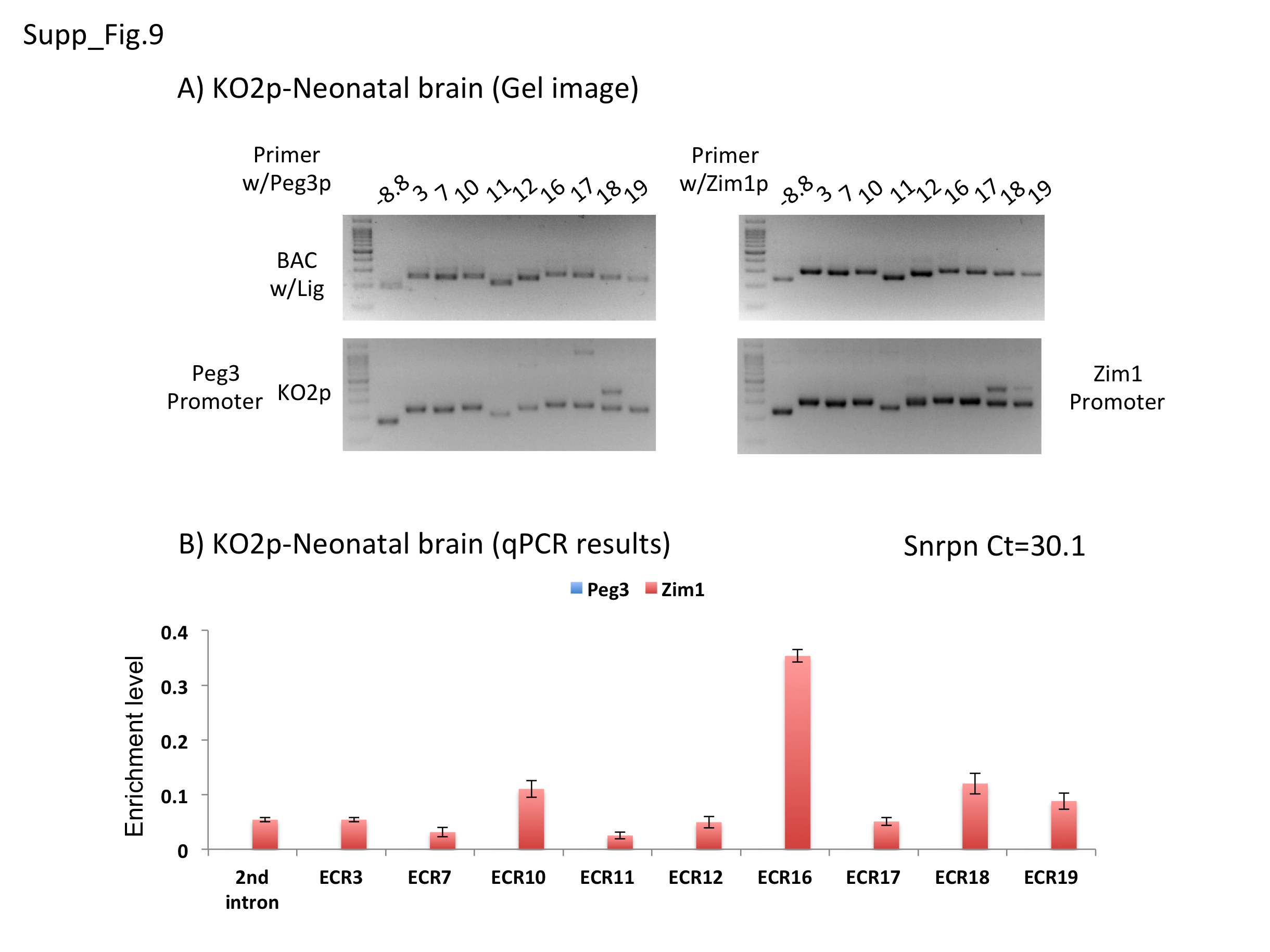

Supplement: S9 File — (TIF) [file pone.0224287.s009.tif]
