# Supplementary material for: Allele-specific enhancer interaction at the Peg3 imprinted domain
Source: PLoS One. 2019 Oct 22;14(10):e0224287. doi: 10.1371/journal.pone.0224287 (PMC6804975; doi:10.1371/journal.pone.0224287)
Supplement: Attachment — Submitted filename: Response_to_reviewer_comments.docx [file pone.0224287.s010.docx]

**Response to Review Comments**

**Reviewer #1**: This is a novel study of gene regulation at the imprinted Peg3 locus using mouse KO lines that have been described in several previous papers from the same group. The mice harbour two different mutations that together switch allelic expression of imprinted genes on the maternal and paternal alleles. Here, by comparing expression, methylation and enhancer/promoter interactions they infer quantitative differences may exist in the interactions between the promoters and distal enhancer regions that are allele-specific.

The data generally look sound and my main advice is that the authors exercise greater caution in the way they choose to interpret their findings. Although they show some quantitative differences to be statistically significant they are relatively subtle. While these differences might be due to allele-specific in promoter/enhancer interactions involving epigenetic changes this remains to be proven. The observed differences could also be a direct consequence of the two different gross changes that have been introduced to the mutant loci. Thus, I would like to see a clear caveat to this effect included in the discussion

------- Response: We have included a sentence describing this potential caveat of the current study in the second paragraph of the Discussion section.

Some specific points:

Introduction (p3, para 1). Is it true to say “The majority of imprinted genes are found only in placental mammals”?

-----Response: Yes, that is correct.

Introduction (p3, para 1). The sentence beginning “The mechanisms through imprinted…” does not make grammatical sense.
------- Response: We have removed that sentence.

Introduction (p3, para 2). In what context is the Peg3 domain “well conserved”, do you mean specifically between mouse and human?
------- Response: The Peg3 domain is well conserved among several mammals, including human, mouse, sheep, dog, and cow.

Introduction (p3, para 2). Reference to Figure 1 would be helpful.
------- Response: We have added a reference pointer to Fig 1 in that paragraph.

Results (p5, para 1). The sentence beginning “The schematic representations…” belongs in the appropriate figure legend.
------- Response: We have moved this sentence to the legend of Fig 1.

Results (p6, para 2). The last line, beginning “on the other hand..” should perhaps refer to expression ‘differences’ being ‘indistinguishable’ rather than ‘levels’ being ‘inconclusive’.
------- Response: We have corrected this as suggested.

Results (p6, para 3). Make clear you are talking specifically about male kidney and male gonadal fat.
------- Response: We have added the term 'male' in the two sentences.

Results (p6, para 3). In the sentence beginning “Although the observed differences..” it is not clear whether the comment “at least ranging from 10 to 100 fold…” refers to brain or non-neural tissues. The whole sentence needs a rethink.
------- Response: We have further clarified this by modifying the sentence.

Results (p7, para 1). “Fourth we analysed the expression levels…” in which tissues etc?
------- Response: We have added the phrase 'in the hypothalamus set.'

Results (p7, para 2). “The results from this series…” Remove this unnecessary statement and others that are similar elsewhere.
------- Response: We put a summary statement at the end of the corresponding paragraph to emphasize the main point of each experiments. We have modified several summary sentences not to be redundant.

Results (p7, para 2). You state that Peg3 expression is ubiquitous in adult brain then later that Zim1 is uniquely expressed in ependymal cells; one of these statements must be wrong.
------- Response: Peg3 is expressed in the majority of neuron cells in the hypothalamus, whereas the expression of Zim1 was more limited to the small areas, such as PVN and SON. Also, we detected high expression levels of Zim1 in the ependymal cells from the current study. Overall, we do not feel these statements are contracting to each other.

Results (p8, para 1). You note that Zim1 expression is “very similar” to Peg3 in PVN and SON. If Peg3 expression is ubiquitous and Zim1 expression more restricted, ZIm1 expression must form a sub-set of Peg3 expression and I don’t see how this forms good evidence they “share long-distance enhancers”. In addition, you specifically indicate enhancers “localized in the middle 200-kb region” of the domain but this cannot be inferred from expression patterns alone.
------- Response: We agree with the reviewer on this point that we need to have additional evidence to support the statements. However, we also strongly believe that the following scenario is most likely. Although Peg3 expression is more ubiquitous than Zim1 expression in the hypothalamus, both genes share a similar spatial expression pattern, the expression within the PVN and SON areas. This unique spatial expression pattern between these two genes is a strong indication that they share a set of cis-regulatory elements, or enhancers. Further, according to the surveys of the ENCODE dataset and also our own results (Kim and Ye 2016), the middle 200-kb region has the majority of potential enhancers within this imprinted domain. Thus, we believe that the shared enhancers are likely localized within the middle 200-kb region.

Results (p8, para 2). Why give a DNA methylation range (“8-37%” for enhancers with low levels but not for those with high methylation levels? You also state that maternal versus paternal differences “appeared to be significant”, but on what basis (statistical test)?
------- Response: We have included the methylation level range of another set of ECRs with high methylation levels. We have also included the p values derived from statistical analyses in the text.

Results (p9, para 2). Not clear that BAC DNA provides a good control for amplification efficiency for 3C experiments involving native cellular DNA.
------- Response: Purified BAC DNA goes through a series of similar digestion and ligation, which is subsequently used for PCR amplification to test the feasibility of a given 3C scheme as well as the amplification efficiency of each amplicon. We believe that this is a standard practice for 3C experiments. However, we also agree with the reviewer that this may not be an ideal control for native cellular DNA.

Results (p9, para 1). I can’t find any primer details for the 3C experiments. Also, why was the Snrpn control chosen, has it been validated previously, perhaps in a prior publication?
------- Response: We are the first group reporting this particular 3C on the Snrpn locus. The information regarding the primers has been included in Supporting information 7. We are also providing an additional set of the detailed information regarding this series of 3C as Supporting information 8.

Results (p10, para 1 and 2). Some potentially important data on Zim1 is ‘not shown’ and there is a lack of quantification until Figure 7. It is not clear to me how this comparison has been done, including the stats, and I am struggling to evaluate its validity.
------- Response: This set of results has been included as Supporting information 9. The results in Fig 7 have been derived by comparing the relative enrichment values of each amplicon or interaction between WT and U1/KO2 animals. The detailed information regarding the raw vales and stats have been included as Supporting information 7.

Discussion (p12, par 2). Four ECRs are indicated (ECR7, 11, 17 and 19), which is different to the 3 highlighted on page 10 of the Results (ECR17, 18 and 19).
------- Response: The ECR18 was not allele-biased but is known to be a target site for MyoD, thus has been included in this statement. We have corrected this mistake.

Discussion (p13, par 1). The section beginning “Interestingly, the pioneer factor MyoD…” and ending with the sentence “Further, this suggests…” comes across as a rather clumsy piece of speculation. I would consider a careful rewrite or omitting this entirely.
------- Response: We have removed this speculative sentence along with two references.

**Reviewer #2**: In the manuscript entitled “Allele-specific enhancer interaction at the Peg3 imprinted domain” by Kim et al. identified putative enhancers localized upstream of Peg3 that displayed allele-biased DNA methylation, and they suggested that some of them participate in allele-biased chromosomal conformations with regional Peg3 and Zim1 promoters and that such long-distance enhancers may contribute to allelic expression of Peg3 and Zim1. In the previous report, the authors reported their interesting double KO mice with switched active alleles of Peg3 and Zim1, that is maternally expressed Peg3 and paternally expressed Zim1 by deletion of both Peg3-DMR (ICR) and oocyte-specific alternative promoter (U1) of Peg3. I agree that their switched mouse is a very good tool for elucidating imprinting mechanism and identification of the paternal-biased ECRs from the Peg3 domain is also a novel and very important finding, however, I don’t think that their results actually support their main conclusion (suggestion) that the long-distance enhancers may contribute to allelic expression of Peg3 and Zim1.

Major comments
1. Imprinted domains are very unique because expression profiles of both of the paternal and maternal alleles are different. Therefore, the authors need to carry out 3C experiment on paternal and maternal alleles separately. The authors insisted that the observed ECR’s interaction with Peg3 and Zim1 are likely allele-specific: Peg3 on the paternal and Zim1 on the maternal allele. However, it is not guaranteed.
In the U1/KO2 mice with maternally expressed Peg3 and paternally expressed Zim1, the interaction of ECR’s and Peg3/Zim1 promoters (Fig. 6B) is far from the reversed version of wild type (Fig. 6A), suggesting that the pattern of interaction between the ECR’s and Peg3/Zim1 promoters is more complex and represents mixed pattern of both of the paternal and maternal interaction. Therefore, they should present the interaction profiles of paternal and maternal alleles in the U1/KO2 case, separately.

------- Response: We are very certain that the interaction of Peg3 with ECRs is paternal-specific and the interaction of Zim1 with ECRs is maternal-specific. In particular, the U1/KO2 sample has only one allele for the Peg3 promoter, which is the maternal allele in this case. Thus, the results from the Peg3 promoter represent the interaction of the maternal allele of Peg3 promoter with ECRs. If the interaction between Peg3 or Zim1 and ECRs is purely based on the promoter strength and associated properties of each gene, but not influenced by ECRs, we should have seen the reversed patterns of 3C results between WT and U1/KO2. However, the results turned out to be far from the reversed patterns, which were also noted by the reviewer. In fact, this has been one of the main evidence supporting our conclusion that ECRs are not biallelic but rather allele-biased.

We also confirmed the allele-specific interaction between Peg3 promoter and ECRs by performing 3C with the samples from the mutant with the paternal deletion of the Peg3 promoter so that the base primer for Peg3 (Peg3p) binds to only one allele, which is the maternal allele. This control experiment did not show any enrichment or interaction, indicating that the repressed maternal allele of Peg3 is not normally available for the interaction with ECRs. This is also the case for Zim1 promoter: the repressed paternal allele of Zim1 promoter is not available for the interaction with ECRs. This set of control experiments has been included as Supporting information 9.

2. The authors suggested that the long-distance enhancers may contribute to allelic expression of Peg3 and Zim1, however, which ones contribute to the change of Peg3/Zim1 allelic expression although I agree that the expression levels of Peg3 and Zim1 are affected by some of the ECR’s by modifying chromosome conformation around Peg3 and Zim1 promoters?

------- Response: We believe that the ones showing allele-biased properties in 3C and DNA methylation should be responsible for the observed differences. So, ECR7, ECR11, ECR17, ECR19 should be the potential candidates.

3. DNA methylation analysis was carried out only using wild type mice but not using U1/KO2 mice. However, it is very important to know the DNA methylation status of the ECR2 and ECR17 in U1/KO2 mice because their methylation difference may cause change of interaction between these ECRs and Peg3/Zim1 promoters.

------- Response: We have tested the majority of ECRs in U1/KO2 and also in U1 and KO2 animals, and the results indicated no major difference in DNA methylation levels of the ECRs in various mutant alleles. Thus, we believe that the DNA methylation levels of ECRs are independent of these mutations.

4. I don’t think that interaction between ECRs and Peg3/Zim1 promoter in Fig. 8 reflects the authors experimental data and their explanation in the text. For one example, why do the fourth type (ECR3, ECR10 and ECR16) tended to show higher enrichment levels on the switched alleles with both promoter (the maternal allele for Peg3 and the paternal allele for Zim1) is classified by biallelic interaction because no enrichment was observed in the wild type (Fig. 6).
------- Response: I agree with the reviewer on this point. We have modified the figure accordingly.

Minor comments
1. Although it is necessary to explain the generation of a mouse mutant (U1/KO2), Figure 1 may be redundant because it’s the same as the previous report (ref. 18). The authors should include the information of the position of U1 promoter in the ECR region.

------- Response: Although redundant, we feel that the current manuscript should stand by itself as a separate unit for the readers, so we have included this particular figure. We have included the position information of U1 relative to the positions of ECRs in the legend of Fig 5.

2. In Fig. 4 (on DNA methylation levels of ECRs), authors described that ECR2 and ECR17 exhibited different levels of DNA methylation between paternal and maternal alleles. However, it is difficult to say that the difference of methylation levels are significant between two alleles because it was provided the data of one sample.

------- Response: We agree with the reviewer on this point. However, given our previous results, the DNA methylation methylation levels seem to be quite stable among biological replicates, since this analysis usually captures the average methylation levels of million cells. Thus, we are very confident that the results presented in this study is likely reproducible.

3. In Figure 3, images of immunostaining are low resolution.
------- Response: The images with higher resolution have been included as Supporting information 4.

**Reviewer #3**: Allele-specific enhancer interaction at the Peg3 imprinted domain
The authors look at the role of ECRs in the control of imprinted gene expression in the Peg3 domain.
They also investigate the consequences of a double switch so the paternal chromosome behaves maternally
and the maternal chromosome behaves paternally.

Major comments.

For the gene expression data shown in Figure 1 only 8 animals appear to have been studied, this is far too few to draw conclusions from and perform meaningful statistical analyses. The error bars shown are for technical replicates - I expect on biological replicates they would be much greater.

------- Response: Yes, the error bars are for technical replicates, but we have repeated each set of 3C experiments with 2 to 3 sets of biological replicates, which have been included as Supporting information 5-7. We are reporting only the results that are consistent and reproducible among these biological replicates.

Are the wildtype samples littermates of the mutants? The expression levels of mutants should be normalised to wildtype littermates to ensure they had the same in utero environment and they are exactly the same age.
------- Response: Yes, the WT animals were all littermates of the U1/KO2 animals.

Also, it is not clear if the genetic background of the chromosome that each deletion is on is the same. Many knockouts are derived on non-C57B/6 strains and the results seen could purely be down to genetic background differences.
------- Response: The U1 allele has been derived from the C57BL/6J background, whereas the KO2 allele from 129/SvJ. The animals with the KO2 allele have been backcrossed more than 10 generations with C57BL/6J. Now, both alleles are on the same genetic background, thus we do not anticipate any artifacts that might be caused by genetic differences.

For the methylation analyses the authors used C57BL/6J females crossed with PWD/PhJ males. However,
for robust methylation analysis reciprocal crosses should be used to eliminate any genetic background effects.
------- Response: Yes, we agree with the reviewer on this point. At the same time, we do not believe that the allelic methylation level differences observed from the two ECRs were caused by genetic background differences based on all the previous results that have been derived from the same cross (Kim et al, 2012; He et al, 2016 and 2017)

In the 3C analysis the authors do not explain how they are able to distinguish the maternal and paternal alleles. Is this through SNPs or are the base primers located within the deletion so they are only interrogating interactions on one chromosome? As this provides a major part of their discussion their methods should be described more fully.
------- Response: The base primer for Peg3 is localized within the deleted region of the KO2 allele, thus the interaction of Peg3 in U1/KO2 was derived from the maternal allele. In the case of WT animals, we are very confident that the interactions of the Peg3 promoter with ECRs were derived from the paternal allele based on the following control experiment. We performed a set of control experiments with the animals with the paternal deletion of KO2, lacking the paternal allele of Peg3 promoter. In this series of control experiments, the interaction of Peg3 promoter with ECRs on the maternal allele was very minimal or marginal, indicating that the repressed maternal allele of Peg3 promoter is not available for the interaction with ECRs. Thus, the interactions of Peg3 with ECRs in the WT animals were mostly derived from the paternal allele. This is also the case for the paternal allele of Zim1 promoter. Thus, we are confident that the 3C experiments with two base primers measure allele-specific interaction with ECRs. This has been included as Supporting information 9.

In Figure 5, the middle panel for the Peg3 promoter wild-type - the band for ECR17 is much larger than expected - why is this?
------- Response: We confirmed later that this band represents the genuine interaction between Peg3 and ECR17, but not some artifactual fragment amplified from an unrelated locus. The different-size product was found to have been amplified due to another NcoI site nearby ECR17.

They mention a further set of 3Cs in KO2 pups lacking the paternal Peg3 allele - these data should be in the supplementary material.
------- Response: We are providing this result as Supporting information 9.

The authors mention that the 3C analyses were repeated twice. The data from all three biological replicates should be combined for the main figures rather that showing them separately. This would allow the statistical analyses to be performed on biological replicates rather than technical replicates (as I assume are being shown in Figure 6).
------- Response: It was not feasible to perform statistical analyses on the combined results of biological replicates, mainly due to the fact that the quality of 3C libraries varied quite a bit among individual biological replicates.

Minor comments

What do the authors mean by "placental mammals". To make it clearer authors should use the accepted
terminology:
Eutherians - mammals that are not marsupials or monotremes
Therians - eutherian and marsupials
------- Response: Yes, we have replaced placental mammals with eutherians.

Line 5 should read eutherian evolution - as only 5 genes are known to be imprinted in marsupials
------- Response: Yes, we have corrected this.

Line 9 should read the majority of imprinted genes are found only in eutherian mammals.
------- Response: Yes, we have corrected this.

Line 10 should read imprinting may have evolved (or emerged) in the therian lineage. - A biological process can not be invented>
------- Response: Yes, we have corrected this.

Line 11 the sentence beginning "The mechanisms through imprinting " needs re-wording as it does not make sense.
------- Response: We have removed this sentence.

In paragraph 3 of the introduction the authors should say that the ECRs are located within the Usp29 transcript.
------- Response: Yes, we have included this information.

The orientation of the region in figures switches from figure to figure which make it difficult for a non-expert in this region to follow what is going on.

------- Response: We have modified Fig 1 to display the consistent orientation of the Peg3 domain as shown in the other figures.
