# Supplementary material for: Allele-specific enhancer interaction at the Peg3 imprinted domain
Source: PLoS One. 2019 Oct 22;14(10):e0224287. doi: 10.1371/journal.pone.0224287 (PMC6804975; doi:10.1371/journal.pone.0224287)
Supplement: Attachment — Submitted filename: Responses_to_revieres_comments_2.docx [file pone.0224287.s011.docx]

**Responses to Reviews' Comments**

**Reviewer #1**: The authors have made efforts to address most of my earlier comments. The few remaining points are as follows:

1. Introduction (p3, para 1). Is it true to say “The majority of imprinted genes are found only in placental mammals”?
Response: Yes, that is correct.
Reviewer reply: I think this needs clarification still. Are you indicating that imprinting is restricted to mammals among animals (which is not contentious) or do you mean to say that most imprinted genes do not have orthologues outside of mammals (which I would contend)?

-----Response: We mean to say that the majority of imprinted genes do not have orthologues outside of mammals.

2. Introduction (p3, para 2). In what context is the Peg3 domain “well conserved”, do you mean specifically between mouse and human?
Response: The Peg3 domain is well conserved among several mammals, including human, mouse, sheep, dog, and cow.
Reviewer response: Please make this clear, e.g. “The Peg3 imprinted domain is localized in 500-kb genomic interval in human chromosome 19q13.4/proximal mouse chromosome 7 that is evolutionarily well conserved among mammals”

-----Response: We mean to say that the Peg3 domain is well conserved among several mammals, including human, mouse, sheep, dog, and cow. We have modified this sentence to further clarify this point.

3. Results (p7, para 2). You state that Peg3 expression is ubiquitous in adult brain then later that Zim1 is uniquely expressed in ependymal cells; one of these statements must be wrong.
Response: Peg3 is expressed in the majority of neuron cells in the hypothalamus, whereas the expression of Zim1 was more limited to the small areas, such as PVN and SON. Also, we detected high expression levels of Zim1 in the ependymal cells from the current study. Overall, we do not feel these statements are contracting to each other.
Reviewer response: Then this is still unclear.

-----Response: Our understanding is that two genes, Peg3 and Zim1, share several enhancers, but each of these two genes can have its own unique expression pattern in specific cell types, as shown in the ependymal cells of the hypothalamus.

4. Results (p8, para 1). You note that Zim1 expression is “very similar” to Peg3 in PVN and SON. If Peg3 expression is ubiquitous and Zim1 expression more restricted, ZIm1 expression must form a sub-set of Peg3 expression and I don’t see how this forms good evidence they “share long-distance enhancers”. In addition, you specifically indicate enhancers “localized in the middle 200-kb region” of the domain but this cannot be inferred from expression patterns alone.
Response: We agree with the reviewer on this point that we need to have additional evidence to support the statements. However, we also strongly believe that the following scenario is most likely. Although Peg3 expression is more ubiquitous than Zim1 expression in the hypothalamus, both genes share a similar spatial expression pattern, the expression within the PVN and SON areas. This unique spatial expression pattern between these two genes is a strong indication that they share a set of cis-regulatory elements, or enhancers. Further, according to the surveys of the ENCODE dataset and also our own results (Kim and Ye 2016), the middle 200-kb region has the majority of potential enhancers within this imprinted domain. Thus, we believe that the shared enhancers are likely localized within the middle 200-kb region.
Reviewer response: Your ‘beliefs’ need to be supported by briefly setting out the logic you have presented here.

-----Response: We have modified this paragraph to make more logical as suggested.

**Reviewer #3**: The authors do not address the point raised about the expression data. In the response they mention 3C experiments rather than expression.

"but we have repeated each set of 3C experiments with 2 to 3 sets of biological replicates, which have been included as Supporting information 5-7. We are reporting only the results that are consistent and reproducible among these biological replicates."

The point being raised was that the expression data from 4 KOs and 4 WTs is not enough and they have not addressed this. Have they repeat the expression data on more individuals? At least 6 animals from each genotype across a number of litters are required. The error bars should then reflect the differences in expression across multiple individuals NOT technical replicates. These data should all be incorporated into 1 graph and shown in Figure 1.

-----Response: We have used these 4 sets of KO and WT derived from the previous breeding experiments, which took place 2 years ago throughout a span of 6 months. This was the maximum number of WT and KO littermate pairs with matching age at that time. Although we understand the reviewer's point, it is not feasible to perform another round of breeding experiments, which may take another 6 month to 1 year. At the same time, we are very confident with the conclusion derived from this set of the results given the associated statistical significances.

I previously asked what was the genetic background the mutations were generated in . This question is not about the background of the genome as a whole but rather the region in close proximity to the deletions. The authors should note that even after backcrossing for 10 generations the DNA in close proximity to the deletion (which is selected for) is unlikely to have had a crossover event and thus will still be 129/SvJ in the case of the KO2 mice. As the KO2 region is close to genes it is highly likely that the genes located on the KO2 chromosome are 129/SvJ. Therefore the U1/KO2 mice Zim1 will be on a SvJ background but in Wildtypes on BL6. Differences in the sequences of genes and cis regulatory elements could explain the subtle differences in expression levels in Zim1.

-----Response: When we first started working on this domain 20 years ago, we have assembled and sequenced two BAC contigs from the 129 and B6 strains. Some of these have been published (Kim et al, 2000 *Genome Research* **10**:1138-1147; Kim et al, 2001 *Genomics* **74**:129-141). According to this survey, we have not found any SNPs between these two strains in the ECRs, promoter and exon regions of the imprinted genes, including Peg3/Usp29 and Zim1. Thus, we believe that the expression difference reported in the current manuscript cannot be caused by the sequence variations between B6 and 129.

On page 9

"The observed ECR's interaction with Peg3 and Zim1 are also likely allele-specific: Peg3 on the paternal and Zim1 on the maternal allele"

This statement is naive: enhancers have been shown to interact with promoters even in tissues where the gene is not expressed (data from Eileen Furlong's lab). The authors show in Supp Fig.9 that upon paternal transmission of KO2 there is reduced interaction of Peg3 promoter with ECRs. But the Zim1 data in this cross is biallelic. To fully characterise the interactions the authors should also perform 3Cseq on maternal KO2 heterozygotes and to interrogate the paternal chromosome separately.

All the data presented in Figure 7 relies on the assumption of allele specific interactions in the WT and U1/KO2 mice but this has not been established in the 3Cs presented. Supp_Figure 9A clearly shows that some interaction occurs between the maternal Peg3 promoter and the ECRs on a gel even though it was not detected in the qPCRs.

In the light of this Figure 7 should be re-labelled to show the mouse genotype, wildtype or U1/KO2, NOT Mat or Pat ECRs to make it clear that these were not allele specific 3Cs.

-----Response: We actually performed a series of 3C using the sample with the maternal transmission of KO2. However, the results cannot be used as a control demonstrating the allele-specific interaction mainly due to the fact that the maternal deletion of KO2 has a very unusual trans-allelic effects, up-regulating the remaining paternal allele (Bretz and Kim, 2018 *PLoS ONE* **13**:e0206112).

Regarding re-labeling Fig 7, both WT and U1/KO2 have their own parental alleles (MAT and PAT). Thus, it will make very busy figures with many labelings. Thus, we included the following sentences in the figure legend. The values for the paternal and maternal alleles of Peg3 were derived from WT and U1/KO2, respectively. On the other hand, the values for the maternal and paternal alleles of Zim1 were derived from WT and U1/KO2, respectively.

All the minor comments have been addressed except on page 3 they should say imprinting emerged in the therian lineage as imprinting has been observed in marsupials too.
